# Dimensionality of joint torques and muscle patterns for reaching

**DOI:** 10.3389/fncom.2014.00024

**Published:** 2014-03-03

**Authors:** Marta Russo, Mattia D'Andola, Alessandro Portone, Francesco Lacquaniti, Andrea d'Avella

**Affiliations:** ^1^Laboratory of Neuromotor Physiology, Santa Lucia FoundationRome, Italy; ^2^Center of Space Biomedicine, University of Rome “Tor Vergata,”Rome, Italy; ^3^Department of Systems Medicine, University of Rome “Tor Vergata,”Rome, Italy

**Keywords:** modularity, reaching movements, human subjects, inverse dynamics, EMGs, muscle synergies, temporal components, joint torques

## Abstract

Muscle activities underlying many motor behaviors can be generated by a small number of basic activation patterns with specific features shared across movement conditions. Such low-dimensionality suggests that the central nervous system (CNS) relies on a modular organization to simplify control. However, the relationship between the dimensionality of muscle patterns and that of joint torques is not fixed, because of redundancy and non-linearity in mapping the former into the latter, and needs to be investigated. We compared the torques acting at four arm joints during fast reaching movements in different directions in the frontal and sagittal planes and the underlying muscle patterns. The dimensionality of the non-gravitational components of torques and muscle patterns in the spatial, temporal, and spatiotemporal domains was estimated by multidimensional decomposition techniques. The spatial organization of torques was captured by two or three generators, indicating that not all the available coordination patterns are employed by the CNS. A single temporal generator with a biphasic profile was identified, generalizing previous observations on a single plane. The number of spatiotemporal generators was equal to the product of the spatial and temporal dimensionalities and their organization was essentially synchronous. Muscle pattern dimensionalities were higher than torques dimensionalities but also higher than the minimum imposed by the inherent non-negativity of muscle activations. The spatiotemporal dimensionality of the muscle patterns was lower than the product of their spatial and temporal dimensionality, indicating the existence of specific asynchronous coordination patterns. Thus, the larger dimensionalities of the muscle patterns may be required for CNS to overcome the non-linearities of the musculoskeletal system and to flexibly generate endpoint trajectories with simple kinematic features using a limited number of building blocks.

## Introduction

How the central nervous system (CNS) coordinates a large number of muscles to generate complex motor behaviors is an open question. The dynamic complexity of the skeletal system with its many degrees of freedom (DoF), the versatility of the motor system, capable of accomplishing many different tasks, and the redundancy and non-linearity of the muscular apparatus all pose challenging control problems. A modular architecture has been proposed as a way for the CNS to tackle the complexity of motor control. In a modular architecture control is subdivided among basic building blocks, allowing for an efficient yet flexible task decomposition. In particular, a modular generation of the muscle patterns might allow for a low-dimensional representation of the motor output incorporating knowledge on the dynamic behavior of the musculoskeletal system into a small set of basic functions shared across tasks and conditions. Recently, the modular control hypothesis has been supported by observations of low-dimensionality in the muscle patterns underlying a variety of motor behaviors in different species. Using multidimensional decomposition techniques such as principal component analysis (PCA), factor analysis (FA), independent component analysis (ICA), and non-negative matrix factorization (NMF) it has been possible to reconstruct the muscle activation patterns as the combination of a small number of components (Tresch et al., 2006; Giszter et al., 2007; Ting and McKay, 2007; Bizzi et al., 2008; Tresch and Jarc, 2009; Lacquaniti et al., 2012; d'Avella and Lacquaniti, 2013). These components may capture different features of the muscle patterns shared across task conditions, such as specific relationships in the strength of activation of groups of muscles, i.e., muscle synergies (Tresch et al., 1999; Ting and Macpherson, 2005) or M-modes (Krishnamoorthy et al., 2003), specific time-courses of the activation waveforms for all muscles, i.e., temporal components (Ivanenko et al., 2004; Dominici et al., 2011), and specific collections of muscle activation waveforms, i.e., time-varying muscle synergies (d'Avella et al., 2003, 2006) but they all construct muscle patterns by linear combinations of a small number of generators. However, even if muscle patterns can be accurately described by such generators, task accomplishment depends on the actual joint torques and the consequent joint motions produced by muscle contractions. Thus, to better understand how motor tasks may be accomplished by the combination of a few muscle pattern generators it is necessary to assess the relationship between the organization of muscle patterns and that of joint torques.

While joint torques underlying many different motor behaviors have been investigated extensively, a characterization of their dimensionality with multidimensional decomposition approaches such as those recently used to analyze muscle patterns is missing. Joint torque generators for a two-joint arm have been identified before using NMF from simulated data (Chhabra and Jacobs, 2006) but not from experimental data. Focusing on reaching movements in vertical planes, as in many previous studies (Soechting and Lacquaniti, 1981; Lacquaniti et al., 1982; Flanders et al., 1994, 1996; d'Avella et al., 2006, 2008, 2011), our aim was to investigate the dimensionality of joint torques and to compare it with the dimensionality of the muscle patterns. Moreover, we wanted to explore systematically the dimensionality of different types of generators, i.e., generators capturing shared structure in the spatial–across joints or muscles–, temporal, and spatiotemporal dimensions. Planar point-to-point reaching movements, for which joint torques can be estimated using a simplified dynamical model of the arm with two joints, are normally associated with bell-shaped velocity profiles and biphasic torque profiles (Morasso, 1981; Soechting and Lacquaniti, 1981). The shape of such profiles is invariant with respect to movement speed (Soechting and Lacquaniti, 1981) or load (Lacquaniti et al., 1982) and the relationship between shoulder and elbow dynamic torques is almost linear (Soechting and Lacquaniti, 1981; Lacquaniti et al., 1986; Gottlieb et al., 1997). Similar observation were made for reaching movements in three-dimensional space (Lacquaniti et al., 1986). These observations indicate that joint torques for reaching have remarkable regularities suggesting that their dimensionality is also low. One might hypothesize that there is a one-to-one relationship between muscle pattern generators and torque generators. However, biomechanical characteristics and constraints must be taken into account.

To generate the joint torques **τ**(*t*) required to move the arm along a given joint trajectory **q**(*t*), i.e., torques for which the trajectory is a solution of the arm motion equations [see Equation (3) in Material and Methods], the CNS, according to the modular control hypothesis, combines a set of *N*_*m*_ (spatial, temporal, or spatiotemporal) muscle pattern generators:

$$m(t)=\sum_{n\;=\;1}^{N_{m}}a_{n}v_{n}(t)$$

where **v**_*n*_(*t*) is the *n*-th spatiotemporal generator or the product of the *n*-th temporal component times the *n*-th spatial weighting vector for spatial and temporal generators (see “Dimensionality of motor commands” in Materials and Methods). The tension generated by the activation of the each muscle is determined by the dynamics of the musculotendon unit, which depends non-linearly on muscle length, velocity, and muscle activation. Muscle length and velocity depend on joint angles and joint velocities via a matrix of moment arms. Then, muscle torque depends on joint angles, joint velocities, and muscle activations

$$\tau =\tau (q,\dot{q},m).$$

Thus, the required torque profile can be generated by appropriate combination of the muscle pattern generators, i.e., it can be expressed as a function of the combination coefficients **a**:

$$\tau =\tau (q,\dot{q},\sum_{n}a_{n}v_{n}).$$

The torque does not depend in general linearly on the muscle activations and, consequently, on the combination coefficients **a**. When linearity is an adequate approximation, muscle torque can be expressed as a linear combination of the “force fields” associated to each generator, **φ**_*n*_ = **φ**_*n*_(**q**, $\dot{q}$) = **τ**(**q**, $\dot{q}$, **e**_*n*_), where **e**_*n*_ is the unit vector along the *n*-th dimension in coefficient space:

$$\tau =\sum_{n}a_{n}\varphi_{n}(q,\dot{q}),$$

i.e., limb control can be achieved by combination of force-field primitives (Bizzi et al., 1991; Giszter et al., 1993, 2007; Mussa-Ivaldi et al., 1994; Kargo and Giszter, 2000a,b; Kargo et al., 2010; Giszter and Hart, 2013). However, torques profiles observed across different task conditions can also be expressed as (or approximated by) a linear combination of *N*_τ_ torque generators:

$$\tau (t)=\sum_{n=1}^{N_{\tau }}b_{n}u_{n}(t).$$

Even if there is a one-to-one relationship between muscle pattern generators and force-field primitives, the number of muscle pattern generators must be larger than the number of torque generators because of the non-negativity constraint on muscle activations. As muscles can only pull, muscle pattern generators are combined with non-negative combination coefficients and, even considering a linear muscle-to-torque mapping, to generate torques spanning a *N*_τ_-dimensional space at least *N*_τ_ + 1 non-negative generators are required (Davis, 1954; Valero-Cuevas, 2009). Moreover, because of the redundancy of the muscular system, different muscle patterns can generate the same torques and thus the muscle pattern dimensionality can be larger than the minimum imposed by non-negativity, i.e., *N*_*m*_ ≥ *N*_τ_ + 1. Thus, the minimum number of muscle pattern generators depends on the actual dimensionality of the joint torque required to perform all conditions of a specific task and the actual number of muscle pattern generators can be larger than the minimum and must be determined experimentally. Importantly, we consider here tasks whose conditions can be described by a set of parameters, such as, for example, the position of a target of a point-to-point reaching movement. Then, since the skeletal system is also redundant for the performance of many tasks, e.g., a specific position of the wrist in space can be achieved with many different joint angle configurations, the actual dimensionality of the joint torques may be lower than the number of joints (i.e., DoF) involved and it must also be determined experimentally. Finally, while the minimal number of muscle pattern generators might guarantee an optimal solution in terms of computational complexity, it might be suboptimal in terms of other costs such as muscular effort. Thus, comparing the torque and muscle pattern dimensionality can provide new information on the control strategy employed to perform a specific task.

We analyzed EMGs data recorded from 19 muscles and kinematic data collected from markers positioned on the arm of subjects performing fast reaching movements from one starting position to 8 targets on the sagittal plane and eight targets on the frontal plane. We used a dynamic model of the arm with four rotational joints (three at the shoulder and one at the elbow) and three translational DoF (the position in space of the shoulder) to estimate joint torques from joint angles with an inverse dynamics computation (Corke, 1996). We then considered the dynamic component of the torques, i.e., the total torques with the gravitational components removed (Gottlieb et al., 1997), and the phasic component of the muscle activity waveforms, i.e., the total rectified and filtered EMG waveforms with the tonic, anti-gravity components removed (Flanders and Herrmann, 1992; d'Avella et al., 2006). Spatial, temporal, and spatiotemporal torque generators were identified by performing PCA on different arrangements of the data matrix. Similarly, spatial, temporal, and spatiotemporal muscle pattern generators were identified with NMF. We first determined the dimensionality of generators according either to a threshold on the fraction of data variation explained (Tresch et al., 1999; Ting and Macpherson, 2005; Torres-Oviedo et al., 2006; Roh et al., 2012) or to the detection of a “knee” in the curve of the variation explained as a function of the number of generators (d'Avella et al., 2003; Cheung et al., 2005; d'Avella et al., 2006; Tresch et al., 2006). We used the former criterion for the torque data and the latter for the EMG data. However, to directly compare the dimensionality of torques and muscle patterns, we then also used a single criterion which took into account the different intrinsic variability of the two datasets when determining their dimensionality (Cheung et al., 2009).

## Materials and methods

### Participants, experimental apparatus, and task

Four right handed subjects (aged between 27 and 40) gave their written informed consent to participate in the study, which conformed with the Declaration of Helsinki and had been approved by the Ethical Review Board of the Santa Lucia Foundation. The experimental apparatus and reaching task has been described in details in a previous report (d'Avella et al., 2006). Briefly, standing subjects gripped with their right hand an handle (weight 180 g) which had a sphere (diameter 4 cm) attached to one extremity. The center of sphere was aligned with the axis of the forearm at a distance of 12 cm from center of the palm. Participants were instructed to move the sphere between a central position and 8 targets uniformly arranged on a circle at 15 cm of distance on either the frontal or sagittal plane while minimizing shoulder and wrist movements. The central position was adjusted for each subject so that it required maintaining the upper arm vertical and aligned to the trunk and the elbow flexed at 90°. The targets were indicated by transparent spheres lighted from inside by an LED. In each trial, after holding the sphere at the start position for at least 1 s, subjects were instructed to move after a go signal, to reach the target with a movement of a duration (defined as the interval in which the speed of the sphere was above 10% of its maximum) shorter than 400 ms, and to hold there for at least 1 s. Unsuccessful trials were repeated. Each subject performed each movement successfully five times in different blocks of trials for a total of 160 point-to-point movements (2 planes × 8 targets × 2 directions -from the center to the target and from the target back to the center- × 5 repetitions).

### Data acquisition

The motion of the arm was recorded using an optic motion-tracking system (Optotrack 3020, Nothern Digital, Waterloo, Ontario, Canada) with a sampling frequency of 120 Hz and spatial resolution below 0.1 mm. Active optical markers were positioned on the shoulder (acromion), the upper arm (at the proximal end close to the head of the humerus), the elbow (epicondylus lateralis), the wrist (one over the styloid process the radius and one on the styloid process of the ulna). The motion of the sphere on the handle (end-point) was recorded with an electromagnetic motion-tracking system (Fastrak, Polhemus, Calchester, VT) with sampling frequency of 120 Hz and spatial resolution below 4 mm, as estimated by a calibration process performed within the workspace used in the experiment.

EMG activity was recorded with active bipolar surface electrodes (DE 2.1; Delsys, Boston,MA) from the following muscles: biceps brachii, short head (BicShort), biceps brachii, long head (BicLong), brachialis (Brac), pronator teres (PronTer), brachioradialis (BrRad), triceps brachii, lateral head (TrLat), triceps brachii, long head (TrLong), triceps brachii, medial head (TrMed) deltoid, anterior (DeltA), deltoid, middle (DeltM), deltoid, posterior (DeltP), pectoralis major, clavicular portion (PectClav), pectoralis major, lower portion (PectLow), trapezius superior (TrapSup), trapezius middle (TrapMid), trapezius inferior (TrapInf), latissimus dorsi (LatDors), teres major (TeresMaj), infraspinatus (InfraSp). EMG signal was band-pass filtered (20–450 Hz) and amplified (total gain 1000, Bagnoli-16, Delsys Inc.). EMG data were digitized at 1 KHz (PCI-6035E, National Instruments, Austin, TX).

Data acquisition and experiment control were performed on a workstation with custom software written in LabView (National Instruments, Austin, TX). Fastrak data were processed on-line to compute the movement time and target accuracy and to provide auditory feedback about unsuccessful trials. The experiment control program logged the time of all relevant behavioral events.

### Data analysis

#### End point kinematics

All analyses were performed with custom software written in Matlab (Mathworks, Natick, MA). Position and orientation of the handle and the measured geometric parameters of the handle were used to compute the position of the end-point. The data were low-pass filtered (FIR filter; 15 Hz cutoff; zero-phase distortion; Matlab *fir1* and *filtfilt* functions) and differentiated to compute tangential velocity and speed. For each movement we computed the *onset time* and the *end time*, defined respectively as the time in which the speed profile crossed 10% of its maximum value, and the *movement duration* (MT), defined as the interval between the movement onset and the movement end.

#### Arm model

A kinematic and kinetic model of the arm, incorporating geometrical and inertial parameters of the upper arm and forearm segments, was used to estimate joint angles and joint torques from the recorded spatial position of the shoulder, the elbow, and the wrist markers. The kinematic model was developed using the Denavit-Hartenberg (D-H) notation (Hartenberg and Denavit, 1955), i.e., as chain of articulated links with four parameters for each link (*a*: length, α: twist, *d:* offset, ϑ: joint angle) describing the position and orientation of a Cartesian reference frame fixed on each link with respect to the reference frame fixed on the preceding link of the chain according to the 4 × 4 homogeneous transformation matrix *T*:

(1) $$T=\;\left[\begin{array}{cccc} \cos\vartheta & -\sin\vartheta \cos\alpha & \sin\vartheta \sin\alpha & a\cos\vartheta \\ \sin\vartheta & \cos\theta \cos\alpha & -\cos\theta \sin\alpha & a\sin\theta \\ 0 & \sin\alpha & \cos\alpha & d\\ 0 & 0 & 0 & 1 \end{array}\right].$$

**Figure 1:**
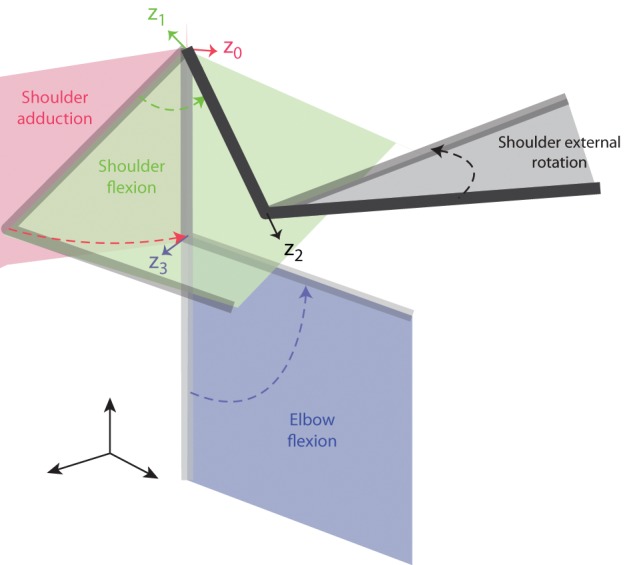
**Joint angle definition for the arm model**. The four joint angles included in the model (shoulder adduction, shoulder flexion, shoulder external rotation, and elbow flexion) are illustrated by a sequence of postures in space of a two-link arm. The four rotation axes are indicated by colored arrows and correspond to the z-axes of the four reference frames defined according to the D-H notation (see text) and labeled *z*_0_–*z*_3_.

The rotation axis of each joint coincides with the *z* axis of the preceding link in the chain. The *x* axis in each frame is directed as the normal between the *z* axis of that frame and the *z* axis of the next frame. In this way the joint angle is the angle between the *x* axes of the frames of the two links connected by the joint. We modeled four rotational degrees-of-freedom (DOFs) of the arm—three rotations at the shoulder, i.e., adduction, flexion and external rotation, and one rotation at the elbow, i.e., elbow flexion (see Figure 1)—and three translational DOFs of the shoulder. We assumed that shoulder was a spherical joint (i.e., the rotation axes of the three joints intersect at a single point). Lengths of upper arm, forearm, and hand of each subject were estimated as a function of the subject's weight and height according to regression equations (Winter, 1990). Forearm, hand, and handle were considered a single link (7th) of length equal to the sum of the forearm length and the length of the opened hand, thus approximating the total length of the closed hand and the handle along the direction of the forearm axis with the length of the opened hand.

The kinetic model of the arm was developed adding to each link its inertial parameters (mass, center of mass, inertia tensor) also estimated as a function of the subject's weight and height according to regression equations (Zatsiorsky and Seluyanov, 1983). No mass was associated to the first three links required to represent the spatial position of the shoulder. However, these translational DOFs were introduced to take into account shoulder movements when estimating the joint torques. The mass of the upper arm was assigned to the 6th link, which had an offset equal to the length of the upper arm segment. The mass of the forearm, hand, and handle was assigned to the 7th link, associated with the elbow flexion. The inertial parameters for this link were computed from the inertial parameters estimated separately from the regression equations for the forearm and hand. As the estimated position of the center of mass of the hand and of the handle coincided, the mass of the handle (180 g) was summed to the mass of the hand. The moments of inertia were computed with respect to its center of mass. The model was implemented in Matlab using the Robotic Toolbox (Corke, 1996, 2011). The D-H parameters of the generic arm model are reported in Table 1 and the specific geometric and inertial parameters estimated for each subject are reported in Table 2.

**Table 1:** **D-H parameters for the 7 DOFs arm model**.

**Link**	**DOF**	**α_*i*_**	**a**_*i*_	**θ_*i*_**	**d_*i*_**	**Type**	**Offset**
1	Sh X	π/2	0	π/2	0	P	0
2	Sh Y	π/2	0	π/2	0	P	0
3	Sh Z	π/2	0	π/2	0	P	0
4	Sh adduction	π/2	0	0	0	R	−π/2
5	Sh flexion	π/2	0	0	0	R	π/2
6	Sh external rotation	π/2	0	0	L_U_	R	π
7	El flexion	0	L_F_	0	0	R	π/2

*Sh, shoulder; El, elbow; L_F_, forearm link length; L_U_, upper arm link length; P is for prismatic joints and R is for revolute joints*.

**Table 2:** **Arm model parameters for individual subjects**.

**Subject**	**1**	**2**	**3**	**4**
Height (cm)	180	162	177	181
Weight (Kg)	84	58	75	78
L_U_ (cm)	33.48	30.13	32.92	33.67
L_F_ (cm)	45.36	40.82	44.60	45.61
rU (cm)	13.91	12.16	13.48	13.78
rF (cm)	26.38	23.57	25.83	26.39
M_U_ (kg)	2.29	1.56	2.03	2.11
M_F_ (kg)	2.00	1.52	1.84	1.90
I(lo) U (kg cm^2^ s^−2^)	46.54	28.54	40.45	42.61
I(ap) U (kg cm^2^ s^−2^)	137.84	74.02	120.09	130.03
I(tr) U (kg cm^2^ s^−2^)	152.50	84.74	133.92	144.65
I(lo) F (kg cm^2^ s^−2^)	21.91	12.93	18.63	19.56
I(ap) F (kg cm^2^ s^−2^)	465.00	295.87	416.54	445.74
I(tr) F (kg cm^2^ s^−2^)	475.79	302.27	425.77	455.45

*L_U_, upper arm length; L_F_, forearm length (including hand and handle); rU, position of the upper arm center-of-mass along the link-6 x axis; rF, position of the upper arm center-of-mass along the link-7 x axis; I(lo) U, inertia along the longitudinal axis of the upper arm; I(ap) U, inertia along the antero-posterior axis of the upper arm; I(tr) U, inertia along the trasversal axis of the upper arm; I(lo) F, inertia along the longitudinal axis of the forearm + hand + handle system; I(ap) F, inertia along the antero-posterior axis of the forearm + hand + handle system; I(tr) F, inertia along the trasversal axis of the forearm + hand + handle system*.

#### Joints kinematics

The arm model was used to estimate at each time sample the shoulder adduction angle, the shoulder flexion angle, the shoulder external rotation angle, the elbow flexion angle using the positions of the shoulder and elbow markers and the mean position between the two wrist markers. For each time sample and each joint angle, a vector between two markers aligned with the axis of the limb segment defining the rotation of that joint (i.e., shoulder and elbow markers for shoulder adduction and shoulder flexion, elbow and wrist markers for shoulder external rotation and elbow flexion) was computed first. Then, the segment vector was transformed into the reference frame associated to the joint according to the matrices defined by Equation (1) and the angle computed as

(2) $$\vartheta_{i}=\tan^{-1}(y/x)$$

where *x* and *y* are the coordinate of the vector in the reference frame associated with the joint rotation axis (*z* axis). To compensate for potential misalignment between the tracker *z* axis and the vertical axis, the coordinates of the markers were first rotated into a Cartesian reference frame with the gravitational acceleration along the *z* axis. The direction of the gravitational acceleration was estimated by means of a calibration of based on tracking two markers attached to the fulcrum and the extremity of a pendulum.

Angular velocity and acceleration were computed by numerical differentiation. To validate the kinematic model, forward kinematics was used to compare estimated and measured end-point trajectories.

#### Inverse dynamics

Joint angles, joint velocities and joint accelerations were used to estimate the torque profiles via recursive Newton-Euler calculation (*rne* function of Matlab Robotics Toolbox). We computed the total torques **τ**

(3) $$\tau =M(q)\ddot{q}+C(q,\dot{q})\dot{q}+G(q)$$

where **M** is the manipulator inertia matrix, **C** is the Coriolis and centripetal torque, and **G** is the gravitational torques. To estimate non-gravitational (dynamic) torques we subtracted gravitational torques from the total torques.

To validate the inverse dynamics calculation we also performed a forward dynamics simulation (*fdyn* function of Matlab Robotics Toolbox) using the arm model and the estimated torque profiles to reproduce the original joint angle trajectories.

#### Data preprocessing

The EMGs for each trial were digitally full-wave rectified, low-pass filtered (FIR filter, 20 Hz cut-off, zero-phase distortion, Matlab *fir1*, and *filtfilt* functions), and integrated over 10 ms intervals. In a few cases muscle waveforms showed some artifacts, possibly due to a partial detachment of the electrode from the skin, or to an high correlation between two or more muscles, and those muscles were removed from further analysis (subject 1: PectLow; subject 2: TrapInf, PectLow).

EMGs and torques for all the trials in each experimental condition (2 planes × 8 targets × 2 directions) were aligned on the time of movement onset and averaged.

Finally, both torques and muscle waveforms were normalized in time to equal MT and resampled with 50 samples per MT. Samples from 0.5 MT before movement onset to 0.5 MT after movement end (total 100 samples) were considered for further analysis.

#### Dimensionality of motor commands

We consider a set of *D* command signals (joint torques or muscle patterns) delivered by a controller in a given time interval (sampled *T* times) to accomplish a task in one of *K* distinct task conditions (e.g., different reaching targets). We hypothesize that a modular controller generates these command signals by modulating and combining a small set of generators whose structure is invariant across all task conditions. The structure of such generators may be defined in the spatial (across signals, i.e., muscles or joints), temporal, and spatiotemporal domains. The dimensionality of the ensemble of command signals is then simply the number of generators necessary to accomplish all *K* tasks conditions.

*Spatial dimensionality* is the number of generators necessary to capture time-invariant relationships between the signals. For *N* generators:

(4) $$x^{k}(t)=\sum_{n=1}^{N}c_{n}^{k}(t)w_{n}$$

where **x**^*k*^(*t*) are the set of signals for condition *k*, i.e., a vector-valued (*D*-dimensional) function time (or a *D* × *T* matrix for discrete time samples), *c*^*k*^_*n*_(*t*) is a condition-dependent, time-varying combination coefficient for the *n*-th generator, **w**_*n*_ is the condition-independent, time-invariant *n*-th spatial generator, i.e., a *D*-dimensional vector capturing the relative activation weight of different signals.

*Temporal dimensionality* is the number of generators necessary to capture temporal components shared across all signals (i.e., space-invariant). For *N* generators:

(5) $$x^{k}(t)=\sum_{n=1}^{N}c_{n}(t)w_{n}^{k}$$

where **x**^*k*^(*t*) are again the set of signals for condition *k*, *c*_*n*_(*t*) is the condition-independent, time-varying *n*-th generator (or temporal component), **w**^*k*^_*n*_ is the condition-dependent, time-invariant *n*-th *D*-dimensional weight vector for the *n*-th component. Notice how the critical difference in the definition of spatial and temporal generators and dimensionality is in the dependence on the task condition (*k*). Indeed, generators are useful concepts only if they can be used for a variety of conditions thus allowing an effective reduction of the number of parameters to select for each condition.

*Spatiotemporal dimensionality* is the number of generators capturing simultaneously invariant spatial and temporal features in the signals. Thus, each generator includes a set of signal components that can be expressed as a time-varying vector. For *N* generators

(6) $$x^{k}(t)=\sum_{n=1}^{N}a_{n}^{k}v_{n}(t)$$

where *a*^*k*^ is a condition-dependent combination coefficient for the *n*-th generator, **v**_*n*_(*t*) is the n-th spatiotemporal generator, i.e., a condition-independent, time-varying *D*-dimensional vector (or a *D* × *T* matrix for discrete time samples). However, as different signals may be related synchronously or asynchronously, we can distinguish the case of *synchronous* spatiotemporal generators:

(7) $$v_{n}(t)=c_{n}(t)w_{n}$$

in which each generator **v**_*n*_(*t*) can be expressed as the product of a scalar function of time *c*_*n*_(*t*)times a time-invariant weight vector **w**_*n*_. In contrast, asynchronous spatiotemporal generators cannot in general be factorized into separate spatial and temporal generators.

In addition to being scaled in amplitude, spatiotemporal generators may also be recruited at different times across task conditions, i.e., they may also show invariance for time shifts (d'Avella et al., 2003, 2006). If we assume that the duration of each spatiotemporal generator is smaller than the duration of the signals, we can incorporate condition-dependent onset times *t*^*k*^_*n*_ into Equation (6):

(8) $$x^{k}(t)=\sum_{n=1}^{N}a_{n}^{k}v_{n}(t-t_{n}^{k})$$

#### Identification of generators and their dimensionality

To investigate the spatial, temporal, and spatiotemporal dimensionality of joint torques and muscle patterns we used multidimensional decomposition techniques to identify the different types of generators. We considered the dynamic component of the torques and the phasic component of the muscle activity waveforms. We then used PCA to identify torque generators and, because of the inherent non-negativity of muscle activity, we used NMF to identify muscle pattern generators. Finally, as discussed below, we selected the number of generators with three different criteria, two specific for each dataset and one for both datasets.

***Dynamic torques and phasic muscle patterns***. Reaching movements in vertical planes require torques and muscle activities to accelerate and decelerate the limb and to balance gravitational forces. In this work we focused on the former components, i.e., dynamic torques and phasic muscle patterns. Dynamic torques were computed as the total torques with the gravitational components [the last term of the right hand side of Equation (3)] removed (Gottlieb et al., 1997). Flanders and collaborators (Flanders and Herrmann, 1992) found that it is possible to distinguish the phasic component (related to the movement) from the tonic one (related to maintain a specific posture of the arm) of an EMG signal. As in d'Avella et al. (2006) we used a subtraction procedure to remove the tonic component, i.e., we subtracted a constant muscle activation level before and after the movement and a linear ramp between the two constant values during the movement. After the subtraction a small fraction of EMG samples assumed negative values, indicating that the phasic EMG activity was lower than the tonic activity. However, in order to use the NMF algorithm, we set to zero all negative values (ratio of negative area over total area of all muscles, 0.15 ± 0.04, mean ±*SD* over subjects). To assess the effect of this procedure on the number of generators and on their structure, we also identified generators from the original phasic muscle patterns without imposing a non-negativity constraint and using an iterative factorization algorithm based on gradient descent in place of NMF (see below).

**Figure 2:**
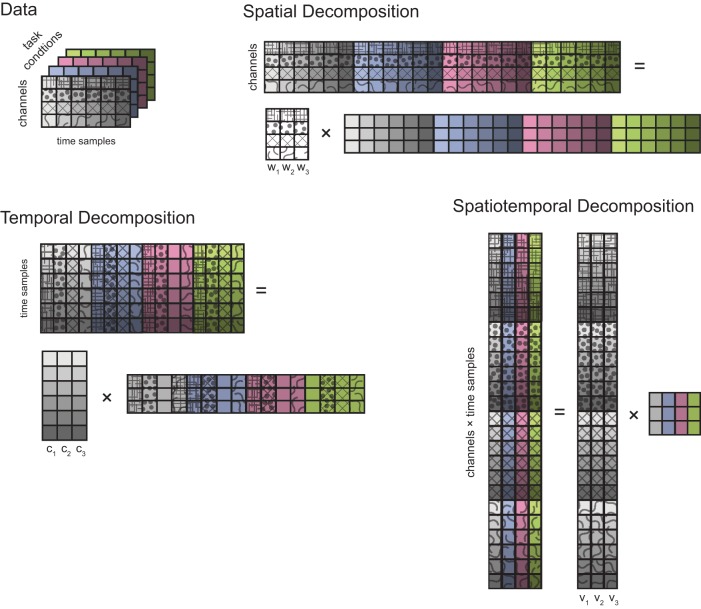
**Types of multidimensional decomposition of data from different task conditions**. Data collected from *D* channels (4 in this schematic illustration, represented by different patterns) over *T* time samples (6, represented by different color saturations) in a single task condition are represented by a grid of squares. Different task conditions are represented with different background colors. A spatial decomposition is obtained by factorizing the data matrix obtained by stacking the data from individual conditions horizontally (i.e., matching their spatial—channels—dimension) into a matrix of *N* (3) spatial generators (*D* rows and *N* columns) times a matrix of time-and condition-dependent coefficients. A temporal decomposition is obtained by factorizing the transpose of data matrix obtained by stacking the data from individual conditions vertically (i.e., matching their temporal dimension) into a matrix of *N* (3) temporal generators (*T* rows and *N* columns) times a matrix of channel- and condition-dependent coefficients. Finally, a spatiotemporal decomposition is obtained by arranging all the data samples of each condition into a column and factorizing the resulting matrix into a matrix of *N* (3) spatiotemporal generators (*D* × *T* rows and N columns) times a matrix of condition-dependent coefficients.

***Data matrices***. To identify spatial, temporal, and spatiotemporal generators, joint torque and muscle patterns data, after pre-processing, were organized into three different data matrices that were factorized by either PCA (torques) or NMF (muscle patterns). For each subjects we identified generators from *K* task conditions (*K* = 32, except for subject 3 for which we had to exclude 2 conditions on the frontal plane and 2 on the sagittal plane because of missing data from the arm markers used to compute joint angles). To identify spatial generators, the data for each condition (*D* signals, EMG or torque, times *T* samples, with *T* = 100 after time normalization and resampling, see Figure 2) were arranged into a data matrix **X** with *D* row and *T* × *K* columns which was factorized, according to Equation (4) in matrix notation, as **X** = **W C**, where **W** is the condition-independent synergy matrix with *D* rows and *N* columns, *N* number of generators, and **C** is the matrix of condition- and time-dependent combination coefficients with *N* rows and *T* × *K* columns. For temporal generators, in contrast, the data matrix was constructed by arranging the waveforms from all signals in all conditions as columns, i.e., **X** had *T* rows and *D* × *K* columns, and it was factorized, according to Equation 5 in matrix notation, as **X** = **C W**, with **C** is the condition-independent matrix of temporal components, with *T* rows and *N* columns, and **W** is the condition- and signal-dependent matrix of weights, with *N* rows and *D* × *K* columns. Finally, for spatiotemporal generators, the data samples for all signals of each conditions were arranged in a column and the data matrix **X**, with *D* × *T* rows and *K* columns, was factorized, according to Equation 6 in matrix form, as **X** = **V A**, with **V** condition-independent matrix of time-varying synergies with *D* × *T* rows and *N* columns and **A** condition-dependent matrix of combination coefficients with *N* rows and *K* columns. For joint torque generators, the covariance of the data matrix was computed and, for each *N*, the first *N* principal components (extracted using MATLAB *pcacov* function) were considered. For muscle pattern generators, for each *N*, **C** and **W** matrices were initialized randomly and the best solution out of 20 runs of the NMF algorithm was selected. Each run of the iterative algorithm was terminated when the reconstruction R^2^ increased in one iteration by less than 10^−4^ for five consecutive iterations. To assess the effect of clipping to zero the negative values of the phasic muscle patterns, we also identified generators without non-negativity constraints using an iterative gradient descent algorithm. We minimized the data reconstruction error by combination of spatial, temporal, and spatiotemporal generators iterating a step in which combination coefficients (**C** and **A**), in the spatial and spatiotemporal cases, and weights (**W**), in the temporal case, were updated and a step in which synergies (**W** and **V**), in the spatial and spatiotemporal cases, and temporal components (**C**), in the temporal case, were updated. Both updates were performed along the direction opposite to the error gradient with step size of 0.05 for the combination coefficients (spatial and spatiotemporal cases), 0.0018 for the weights (temporal case), 0.0005 for the synergies (spatial and spatiotemporal cases), 0.0019 for the temporal components. These gradient step sizes were selected within a range of values in order to achieve the highest reconstruction R^2^. As in d'Avella et al. (2006), in the spatial and spatiotemporal cases, we added a term to the error function to penalize large negative values in the identified synergies. The same number of runs and termination conditions as for the NMF algorithm were used.

***Selection of the number of generators***. For torque generators, we selected their number as the minimum number which explained at least 90% of the data variation (VAF or R^2^, defined as 1—SSE/SST, with SSE sum of square residuals of the data reconstruction by the generators, and SST sum of the squared residuals of the data with respect to the mean over the rows of the data matrix). Such criterion (“R^2^ threshold”) has been frequently used in the muscle synergy literature (Tresch et al., 1999; Ting and Macpherson, 2005; Torres-Oviedo et al., 2006; Roh et al., 2012), even if sometimes with a different definition (i.e., with SST defined as the sum of the squared data, see Delis et al., 2013). Such criterion is based on the assumption that the fraction of data variation unexplained is due to noise and the threshold is supposed to separate structured variation due to the combination of generators and noise. However, if an independent estimation of the noise level is not available the choice of such threshold is necessarily *ad-hoc*. An alternative approach, also used in previous studies (d'Avella et al., 2003; Cheung et al., 2005; Tresch et al., 2006), that we used for selecting the number of muscle pattern generators is the detection of a “knee” in the curve of R^2^ as a function of the number of generators. Such criterion (“R^2^ knee”) relies on the assumption that the noise is isotropic, i.e., contributes equally to all dimensions, and does not depend on a specific assumption of the relative level of noise. To detect a change in slope in the R^2^ curve, for each *N*, we performed a linear fit of the portion of the curve from *N* to the end (i.e., *D*) and we selected *N* for which the mean square error of the fit was <10^−4^, indicating that the “tail” of the curve after the “knee” was essentially straight. We could not use this second criterion for the torques as their maximum spatial dimension (4) was too low and it was impossible to identify a “knee” with such procedure. However, to compare torque and muscle pattern with the same criterion, we also determined their dimensionality with a criterion (“R^2^ shuffle”) that took into account the different intrinsic noise levels of the two datasets. We then used a threshold on the slope of the R^2^ curve according to slope of the curve obtained after a random shuffling the rows of the data matrix (Cheung et al., 2009). Data samples after shuffling were low-pass filtered to match the smoothness in the original data. By shuffling the data the multidimensional structure of the original data was lost but each dimension maintained the original variability. Thus, we selected the number of generators as the point on the original R^2^ curve at which any further increase in the number of extracted generators yielded an R^2^ increase smaller than 75% of that for the generators extracted from the shuffled data (mean over 50 extractions from reshuffled data).

***Comparison of generators across subjects***. To compare generators across subjects, we tested how well a set of generators identified in one subject could reconstruct the data of a different subject. We then computed a *R*^2^-value to assess the similarity of the subspaces spanned by the generators of different subjects. To assess the significance of these *R*^2^-values we performed a Monte Carlo simulation identifying generators from random data obtained by randomly shuffling the original data (50 runs for each subject and type of generator). We then computed the 95% percentile of the distribution of *R*^2^-values for the reconstruction of the original data with generators identified from random data.

***Effect of potential contamination of EMG recordings by cross-talk***. As surface EMG recordings can be affected by cross-talk due to volume conduction of the EMG signal from neighboring muscles, we performed a Monte Carlo simulation to assess the effect of such potential contamination on the dimensionality of the spatial muscle pattern generators. For each muscle, i.e., the *i*-th row of the normalized data matrix **X**, we simulated a cross-talk contamination from a second muscle (*j*-th row), randomly chosen from all other muscles and not limited to the neighboring ones, according to a cross-talk weight (α) randomly drawn from an exponential distribution with mean 0.1, i.e., X′_ik_ = X_ik_ + α X_jk_. We then identified the generators from the contaminated data matrix **X**′ with NMF and we estimated their dimensionality. For each subject, we performed a total of 100 simulation runs, and, across all subjects, we found that the dimensionality estimated using the contaminated data matrix was different from the dimensionality estimated using the original matrix only in 6.7% of runs (0 runs for subjects 1, 2, 4; 27 runs for subject 3, mean dimensionality difference 0.27). Thus, while we cannot exclude that our EMG recordings were not affected by cross-talk, we are confident that such potential contamination did not significantly alter muscle pattern dimensionality estimation.

## Results

### Dynamic torques

**Figure 3:**
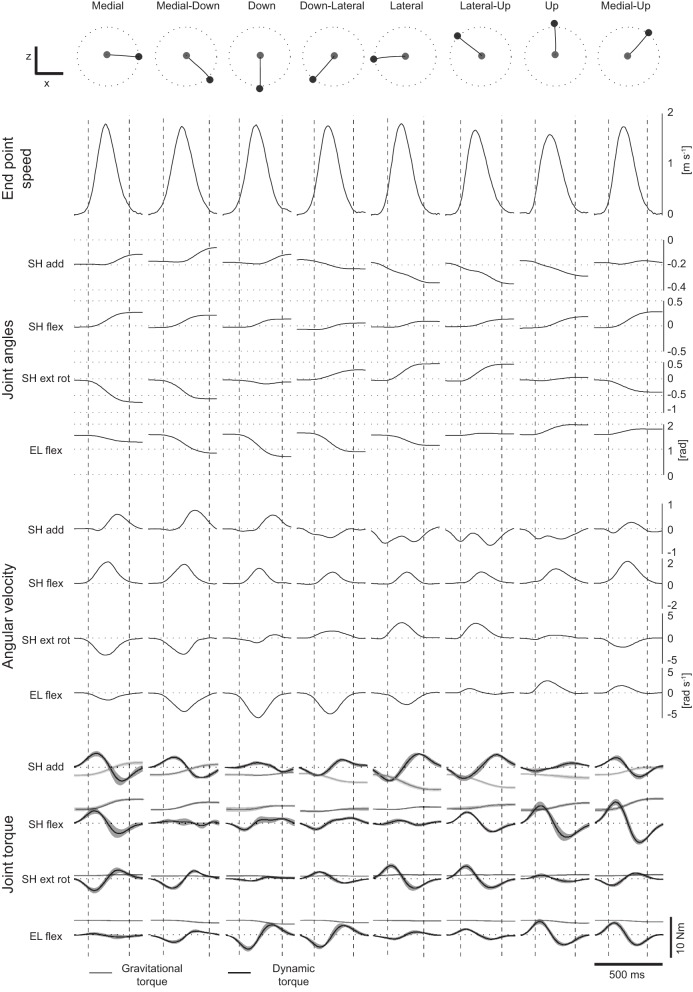
**Example of endpoint speed, velocity, joint angles and torques**. Example of endpoint trajectories, end-point speed profiles, joint angles, joint angular velocities, gravitational (*light gray*) and dynamic (*dark gray*) torques for eight center-out movements in the frontal plane of subject 1 (mean across repetitions of each movement). Vertical dashed lines represent the times of movement onset and movement end. Shaded areas around gravitational and dynamic torque profiles represent ±1 *SD* around the mean.

**Figure 4:**
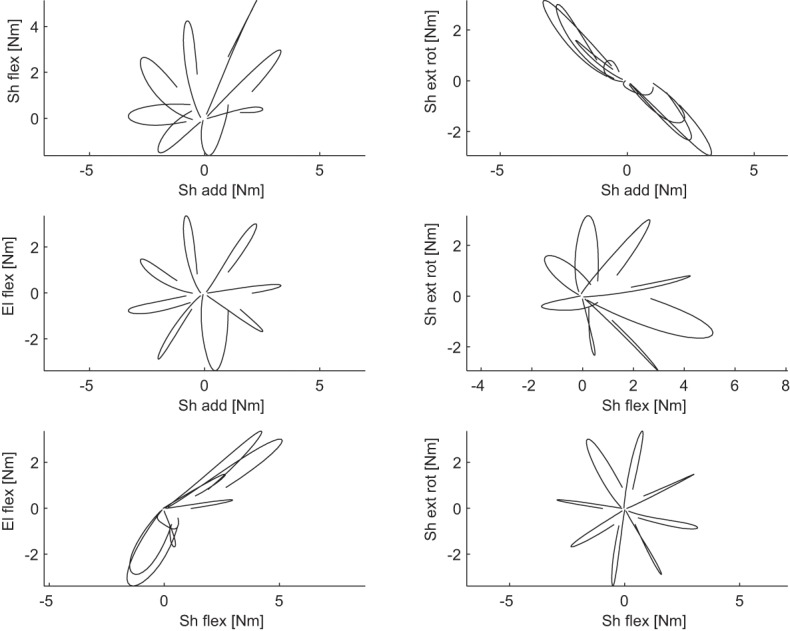
**Example of coordination between pairs of dynamic torques**. Each scatter plot illustrated the dynamic torques for a pair of joints recorded in an interval of 250 ms around the time of movement onset for 8 center-out movements in the frontal plane of subject 1.

Joint torques were estimated by inverse dynamics from joint angle trajectories using a kinetic model of the arm parametrized by the height and weight of each subject. To validate the arm model and the results of the inverse dynamics computation we performed a forward dynamic simulation. The joint angle trajectories simulated using the torques estimated by inverse dynamics matched well the original joint angle trajectories of all subjects and conditions (*R*^2^ = 0.99 ± 0.02, mean across subjects ±*SD*). Figure 3 shows an example of end point trajectories, end point speed profiles, joint angle trajectories, angular velocities, and gravitational and dynamic joint torque profiles for eight center-out movements on the frontal plane. As expected, end point trajectories were straight and velocity profiles bell-shaped. Joint angle trajectories and the corresponding angular velocities were modulated by movement direction. Dynamic torques, i.e., total torque with the gravitational torques removed, were bi-phasic, as observed before (Gottlieb et al., 1997). The time courses of the joint angle trajectories and angular velocities were different across joints and conditions but, because of the dynamic interaction between the different DOFs and they were generated by a synchronous biphasic pulse of torque distributed across joints with different balances depending on the movement direction. Such coordination patterns in the dynamic torque profiles is clearly visible in a scatter plot of a pair of joint torques. Figure 4 shows the six scatter plots of all pairs of joint torque profiles, during an interval of 250 ms around movement onset, approximately capturing the first phase of the profile, for the same 8 movements of Figure 3. If a pairs of torques were modulated synchronously, the corresponding trajectory in the scatter plot would appear as a straight line segment with a direction depending on the relative amplitude. Indeed, for most pairs and movement directions dynamic torques appeared to be modulated close to synchronously, especially in the initial (raising) portion of the profile. Finally, for two pairs of dynamic torques, shoulder external rotation-shoulder adduction and elbow flexion-shoulder flexion, the direction of the line segment in the scatter plot depended only weakly on the movement direction, suggesting that the dynamic torques were spanning a subspace of the four dimensional torque space orthogonal to those two directions. We then generalized these observations by identifying dynamic torque generators and estimating their dimensionality.

#### Spatial dimensionality

We first assessed the spatial dimensionality of the dynamic torques by identifying spatial generators, i.e., vectors in the torque space capturing specific balances of torque magnitude which could reconstruct the data once multiplied by time- and condition-dependent coefficients (see Materials and Methods and Figure 2), using PCA. For each subject, the number of generators was selected as the minimum number for which the fraction of data variation explained exceeded 0.9 (“R^2^ threshold” criterion) and as the number of generators for which adding an additional generator increased the *R*^2^-value less than 75% of the mean *R*^2^-values obtained identifying generators from shuffled data (“R^2^ shuffle” criterion). The mean dimensionality across subjects was 2.25 according to the R^2^ threshold criterion and 2.75 according to the R^2^ shuffle criterion (see Table 3 for individual values). The maximum potential spatial dimensionality of the torques was 4, corresponding to the number of joints, i.e., the number of rows of the data matrix used for spatial decomposition (Figure 2).

**Table 3:** **Comparison of different types of dimensionality of dynamic torques and phasic muscle patterns estimated according to three criteria for the selection of the number of generators**.

**Subject**	**Criterion**	**Torques**			**Muscle patterns**		
		**Spatial**	**Temporal**	**Spatiotemporal**	**Spatial**	**Temporal**	**Spatiotemporal**
S 1	R^2^ threshold	2	1	3	–	–	–
	R^2^ knee	–	–	–	4	5	5
	R^2^ shuffle	3	1	3	4	4	7
S 2	R^2^ threshold	3	1	3	–	–	–
	R^2^ knee	–	–	–	5	5	6
	R^2^ shuffle	3	1	3	5	4	7
S 3	R^2^ threshold	2	1	3	–	–	–
	R^2^ knee	–	–	–	6	4	6
	R^2^ shuffle	3	1	3	6	3	7
S 4	R^2^ threshold	2	1	2	–	–	–
	R^2^ knee	–	–	–	5	4	5
	R^2^ shuffle	2	1	2	5	4	8

**Figure 5:**
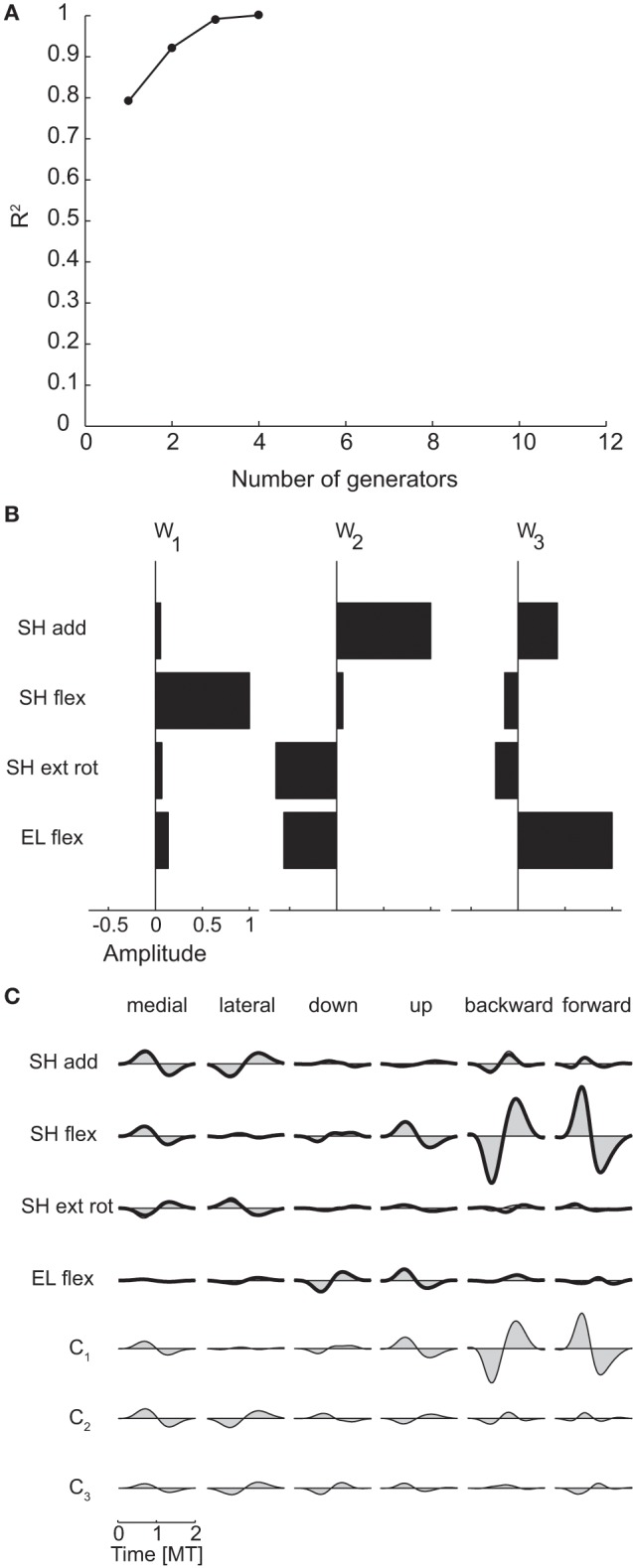
**Spatial decomposition of dynamic torques. (A)** R^2^ curve for subject 1 obtained by spatial decomposition using PCA. **(B)** Three spatial generators selected for subject 1. **(C)** Example of the reconstructions of the dynamic torques for six movement conditions of subject 1 obtained with the generators illustrated in panel **(B)** (*shaded area*: original data, *thick line*: reconstructed data, *bottom*: time-varying combination coefficients).

Figure 5A shows the *R*^2^-value as a function of the number of generators for subject 1 and Figure 5B the three spatial generators of the same subject selected according to the R^2^ shuffle criterion. The first generator (**w**_**1**_) is dominated by shoulder flexion torque. The second generator (**w**_**2**_) combines a large shoulder adduction torque with a smaller shoulder internal rotation (i.e., negative external rotation) and elbow extension (i.e., negative elbow flexion). Finally, the third generator (**w**_**3**_) represents a large elbow flexion torque and a smaller shoulder adduction torque. Notably, none of the generators or their combinations can generate coordinated shoulder adduction and shoulder external rotation torques, i.e., the direction orthogonal to the torques direction observed in the corresponding scatter plot of Figure 4. Thus, the structure of the spatial generators indicated that such torque coordination pattern was never used to perform reaching movements in the frontal and sagittal planes.

Figure 5C illustrates an example of the reconstruction of the dynamic torque profiles of subject 1 in six different conditions by the combination of the three spatial generators of Figure 5B. The dynamic torques for the first two conditions, medial and lateral movements in the frontal plane, are generated by a comparable level of activation of all three generators with a bi-phasic activation of shoulder adduction and internal rotation followed by shoulder abduction and external rotation for the medial movement and the opposite order for the lateral movement captured mainly by the activation of the second generation with similar bi-phasic profiles but opposite signs of its combination coefficient (c_2_). The last two conditions, backward and forward movements in the sagittal plane, require large shoulder flexion/extension torques that are generated by a bi-phasic activation of the first generator, captured by the first time-varying coefficient (c_1_).

To assess the similarity between the subspaces spanned by the generators identified in each subject we reconstructed all dynamic torques of each subject with the generators of all subjects. Table 4 shows the *R*^2^-values obtained using the number of generators determined according to the R^2^ shuffle criterion (see Table 3). The *R*^2^-values for the reconstruction of the data of each subject by the generators extracted from the other subjects (0.96 ± 0.04, mean ±*SD*, *n* = 12) were close to the *R*^2^-values of the reconstruction of the data of each subject by the generators extracted from the same data (0.98 ± 0.02, *n* = 4) and were significantly higher than the *R*^2^-values obtained with generators identified from randomly shuffled data, indicating that the dynamic torques of the different subjects shared a similar spatial organization.

**Table 4:** **R^2^ for the reconstruction of the data of each subject with the torque generators identified in all subjects**.

	**Generators/data**	**S 1**	**S 2**	**S 3**	**S 4**
Spatial	S 1	0.99	0.99	0.98	0.99
	S 2	0.99	0.99	0.97	0.99
	S 3	0.99	0.98	0.99	0.99
	S 4	0.92	0.90	0.88	0.95
Temporal	S 1	0.95	0.92	0.92	0.93
	S 2	0.94	0.93	0.94	0.93
	S 3	0.91	0.92	0.94	0.92
	S 4	0.94	0.92	0.93	0.93
Spatiotemporal	S 1	0.97	0.92	0.90	0.94
	S 2	0.94	0.95	0.92	0.93
	S 3	0.90	0.89	0.96	0.92
	S 4	0.88	0.84	0.82	0.91

#### Temporal dimensionality

To identify generators of the temporal organization of dynamic torques we performed PCA on the collection of the torque profiles of all joints and conditions. The resulting temporal components were then waveforms with the same duration as the torque profiles and each profile was reconstructed by multiplying the component matrix by a weight specific for that joint and condition. The dimensionality was 1 for all subjects and for both criteria (see Table 3). In contrast, the maximum potential temporal dimensionality of the torques was 100, corresponding to the number of time samples after time-normalization and resampling from −0.5 MT before movement onset and 0.5 MT after movement end, i.e., the number of rows of the data matrix used for temporal decomposition (Figure 2).

**Figure 6:**
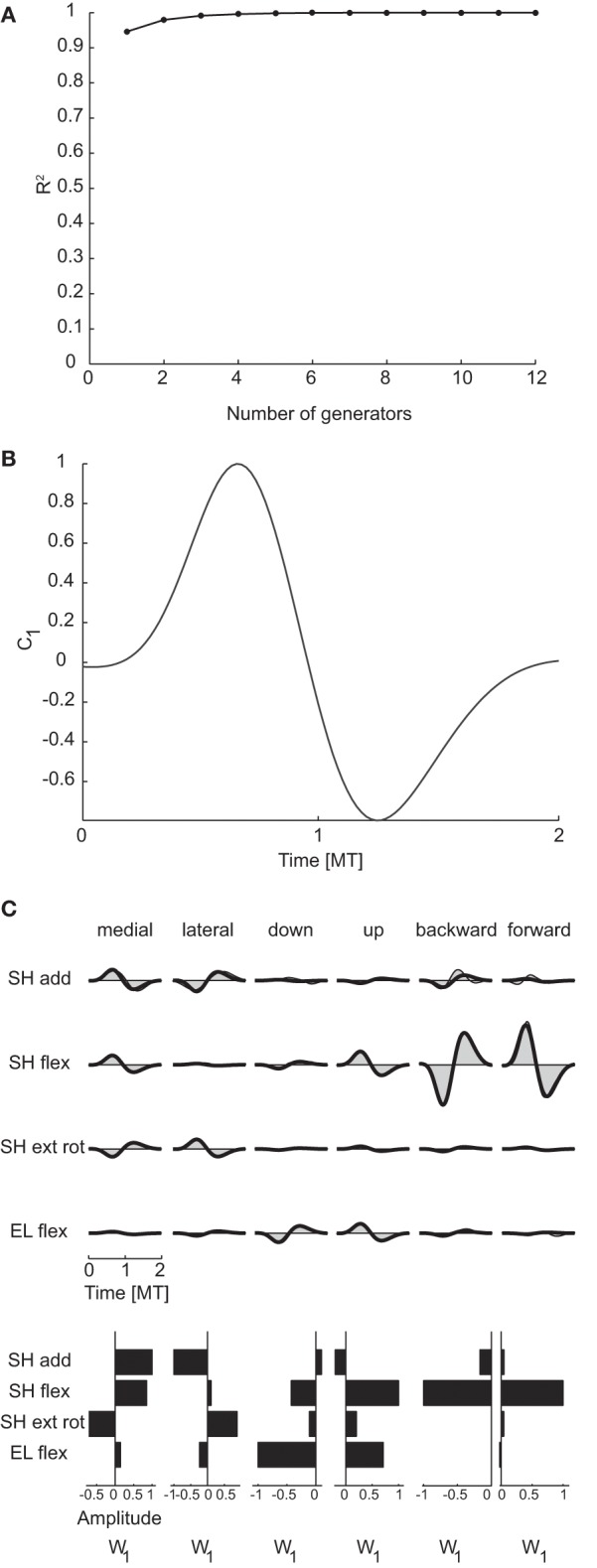
**Temporal decomposition of dynamic torques. (A)** R^2^ curve for subject 1 obtained by temporal decomposition using PCA. **(B)** The single temporal generators selected for subject 1. **(C)** Example of the reconstructions of the dynamic torques for six movement conditions of subject 1 obtained with the generator illustrated in panel **(B)** (*shaded area*: original data, *thick line*: reconstructed data, *bottom*: joint-specific weights).

Figure 6A illustrates the R^2^ curve for the temporal decomposition up to 12 generators for subject 1 and Figure 6B the single temporal component identified in this subject and representative of all subjects, clearly showing a bi-phasic profile. Figure 6C illustrates the reconstruction of the joint torques for the same six conditions of Figure 5C by the temporal generator. The torque profiles for each condition are reconstructed multiplying the single temporal component (c_1_) by a single condition-dependent weight vector (**w**_**1**_). With respect to the reconstruction with spatial generators, the weight vector, which has the same dimensions of a spatial generator, is now modulated by the movement. For example the opposite signs in the bi-phasic profiles of shoulder adduction and external rotation for medial and lateral movements and for shoulder flexion for backward and forward movements are obtained by opposite signs of the components of the weight vector.

Finally, the temporal generators were also similar across all subjects. As for spatial generators, the reconstruction of the data of each subject by the generators of all other subjects had *R*^2^-values (0.93 ± 0.01, mean ±*SD*) which were comparable with the *R*^2^-values for the reconstruction of the data of each subject by the generator extracted from the same data (0.94 ± 0.01) and significantly higher than the *R*^2^-values obtained with generators identified from randomly shuffled data.

#### Spatiotemporal dimensionality

**Figure 7:**
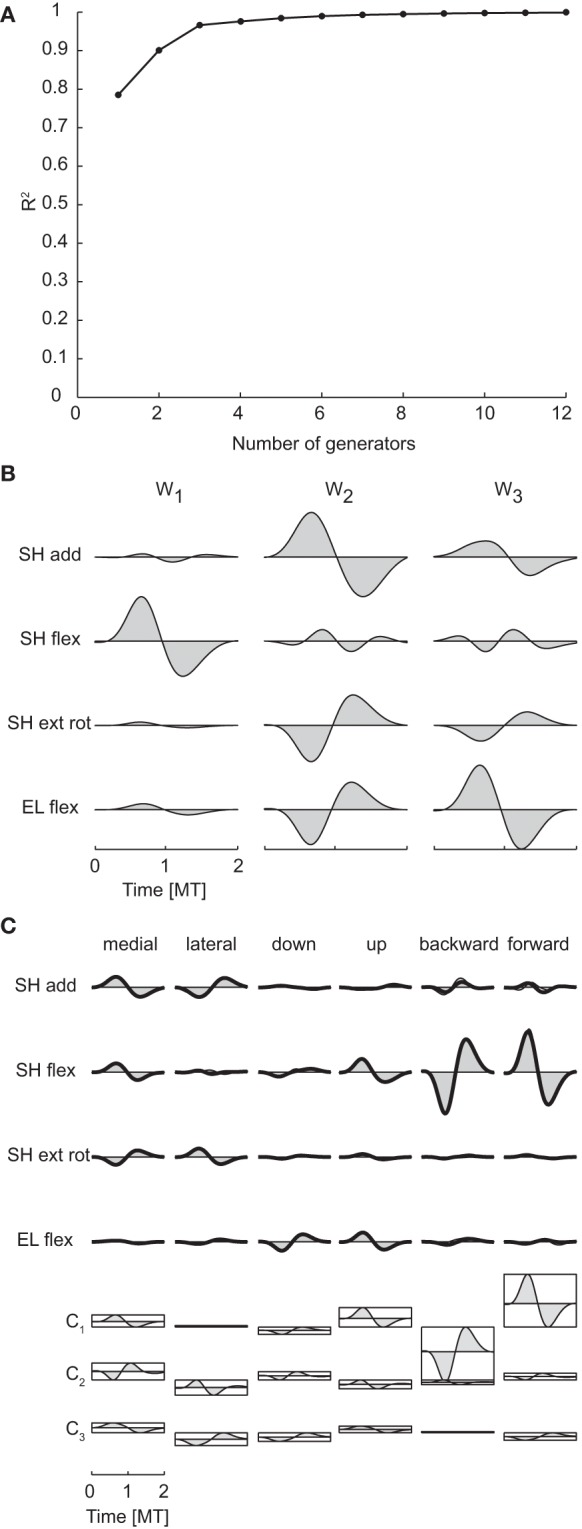
**Spatiotemporal decomposition of dynamic torques. (A)** R^2^ curve for subject 1 obtained by spatiotemporal decomposition using PCA. **(B)** Three spatiotemporal generators selected for subject 1. **(C)** Example of the reconstructions of the dynamic torques for six movement conditions of subject 1 obtained with the generator illustrated in panel **(B)** (*shaded area*: original data, *thick line*: reconstructed data, *bottom*: combination coefficients represented by the height of the rectangle containing the temporal profile of each generators averaged over joints).

Spatiotemporal generators, which can be viewed as either time-varying vectors capturing a different spatial coordination among torques at each time or as collections of different waveforms for each torque, were identified by PCA on a data matrix obtained arranging all time samples from all joints in a single column for each movement condition. Thus, torque samples from different joints and times represented different dimensions and the possibility of generating the data with a number of generators smaller than the maximum potential dimension (400, corresponding to the number of joints times the number of samples) revealed a coordination in the activation of different joints at different times. Once a set of spatiotemporal generators are identified, the data are reconstructed by multiplying each generator by a single condition-dependent coefficient (see Figure 2). Thus the spatiotemporal decomposition provides a potentially very compact representation of the structure inherent in the data. The mean spatiotemporal dimensionality across subjects was 2.75 according to both criteria (see Table 3 for individual values). Notably, mean spatial and spatiotemporal dimensionalities were very close and even equal for each subject when considering the R^2^ shuffle criterion. Moreover, as the temporal dimensionality was 1, the spatiotemporal dimensionality was essentially the product of the spatial and the temporal dimensionalities.

pt Figure 7A illustrates the R^2^ curve for the spatiotemporal decomposition up to 12 generators and Figure 7B the three spatiotemporal component for subject 1. Comparing the structure of these generators with that of the spatial (Figure 5B) and temporal (Figure 6B) generators of the same subject, it is apparent how each spatiotemporal generator appears as the product of a spatial generator by a temporal one. Indeed, the activation waveforms of all spatiotemporal generators are approximately synchronous and similar to the waveform of the single temporal generator. Figure 7C illustrates the reconstruction of the joint torques for the same six conditions of Figures 5C, 6C. The torque profiles for each condition are reconstructed multiplying each spatiotemporal component by a single condition-dependent coefficient (**c**_**i**_), represented by the height of the rectangle below the torque profiles. With respect to the reconstruction with spatial and temporal generators, movements requiring torque profiles with opposite signs are generated simply by changing the sign in a single combination coefficient, e.g., c_2_ for medial and lateral movements and c_1_ for backward and forward movements.

As in previous cases, spatiotemporal generators were similar across subjects. The *R*^2^-values for the reconstruction of the data of each subject by the generators of all other subjects (0.90 ± 0.04, mean ±*SD*) were close to the *R*^2^-values for the reconstruction of the data of each subject by the generator extracted from the same data (0.95 ± 0.02) and significantly higher than the *R*^2^-values obtained with generators identified from randomly shuffled data.

#### Potential misestimation of subject mass and height

The estimation of the joint torques from the recorded joint kinematics through the inverse dynamic calculation depends on geometric and inertial parameters which are estimated as a function of the subject mass and height according to anthropometric tables (see Arm Model section in Materials and Methods). We assessed the effect of a potential misestimation of such parameters on the estimated torque dimensionality by identifying joint torque generators after varying the mass and the height of each subject by ±5, ±10, ±15, ±20%. We recomputed joint torques for individual trials of all subjects and re-processed the torque data as with the original parameters. Across all subjects and types of generators, the dimensionality was affected by a change in mass in 8 out of 96 cases (4 subjects × 3 types of generators × 8 mass change levels) and by a change in height in nine cases. Thus, the estimation of torque dimensionality is robust to small errors in the estimation of anthropometric parameters.

### Muscle patterns

Phasic muscle patterns, obtained by subtracting the anti-gravity (tonic) components from the rectified, filtered, averaged, time-normalized, and resampled EMG waveforms, were decomposed with NMF to assess their dimensionality. Phasic muscle patterns for fast reaching movements in vertical planes have been described before (d'Avella et al., 2006). In contrast to our previous study, here we identified spatial generators, temporal generators, and spatiotemporal generators without onset delays and we compared their dimensionality with the dimensionality of the corresponding generators of dynamic torques.

#### Spatial dimensionality

The mean spatial dimensionality of the phasic muscle patterns across subjects was five according to the position of change in slope of the R^2^ curve as a function of the number of generators (R^2^ knee criterion) and five according to the R^2^ shuffle criterion (see Table 3 for individual values). Thus, as expected, the dimensionality of the muscle pattern generator was larger than the number of spatial torque generators (2.75 according R^2^ shuffle criterion) as muscle pattern generators could only be combined with non-negative time- and condition-dependent combination coefficients. However, the number of muscle pattern generators was larger than the minimum required for generating a space of the same number of linear dimensions as the torque generators (2.75) by non-negative combinations (3.75 = 2.75 + 1).

**Figure 8:**
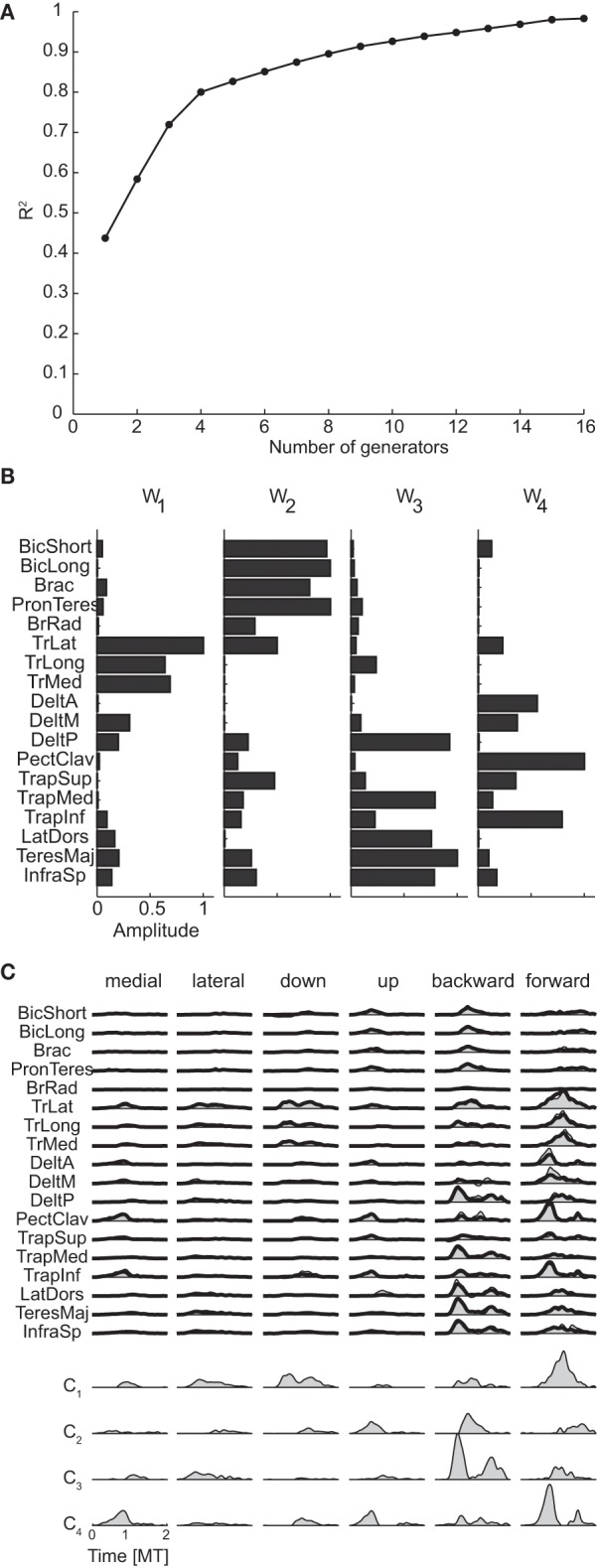
**Spatial decomposition of phasic muscle patterns. (A)** R^2^ curve for subject 1 obtained by spatial decomposition using NMF. **(B)** Four spatial generators selected for subject 1. **(C)** Example of the reconstructions of the muscle patterns for six movement conditions of subject 1 obtained with the generators illustrated in panel **(B)** (*shaded area*: original data, *thick line*: reconstructed data, *bottom*: time-varying combination coefficients).

Figure 8A shows the R^2^ curve for the spatial decomposition of the phasic muscle patterns of subject 1, in which a knee at four generators is clearly visible. The lower *R*^2^-value at the selected number of muscle patterns generators (0.80) with respect to the corresponding value for the torque generators (0.99) indicated that a much larger fraction of the muscle data variation was due to noise. The four spatial generators (or time-invariant muscle synergies) for the same subject illustrated in Figure 8B (**w**_**1**_–**w**_**4**_) show specific groupings of muscles spanning multiple joints and with the same muscle recruited by multiple generators. Finally, in Figure 8C the examples of the reconstruction of the phasic muscle patterns for six movement conditions by the combination of the spatial generators are presented. The temporal structure of muscle patterns and of combination coefficients is clearly more complex than that of the spatial generators with the tri-phasic organization of the muscle patterns generated by both the temporal structure of the combination coefficients and by the superposition of different generators.

Finally the spatial generators for the muscle patterns were less similar across subjects than the spatial generators for the torques (see Table 5). The reconstruction of the data of each subject by the generators of all other subjects had a *R*^2^-values (0.66 ± 0.06, mean ±*SD*) much lower than the mean R^2^ for the reconstruction by the generators extracted from the same data (0.80 ± 0.04) but still significantly higher than the *R*^2^-values obtained with generators identified from randomly shuffled data.

**Table 5:** **R^2^ for the reconstruction of the data of each subject with the muscle pattern generators identified in all subjects**.

	**Generators/data**	**S 1**	**S 2**	**S 3**	**S 4**
Spatial	S 1	0.80	0.60	0.54	0.62
	S 2	0.64	0.74	0.61	0.70
	S 3	0.67	0.71	0.80	0.69
	S 4	0.73	0.75	0.60	0.85
Temporal	S 1	0.88	0.83	0.88	0.83
	S 2	0.85	0.86	0.88	0.85
	S 3	0.81	0.79	0.86	0.79
	S 4	0.77	0.78	0.83	0.81
Spatiotemporal	S 1	0.84	0.30	0.27	0.32
	S 2	0.37	0.77	0.41	0.51
	S 3	0.25	0.41	0.79	0.36
	S 4	0.38	0.49	0.39	0.80

#### Temporal dimensionality

The mean number of temporal generators of the phasic muscle patterns was 4.5 according to the R^2^ knee criterion and 3.75 according to the R^2^ shuffle criterion (see Table 3 for individual values). As for the spatial generators, the temporal dimensionality of the muscle pattern generators was larger than the minimum number required to generate a space with the same linear dimensions as the number of temporal torque generators (1) by non-negative combinations (2 = 1 + 1).

**Figure 9:**
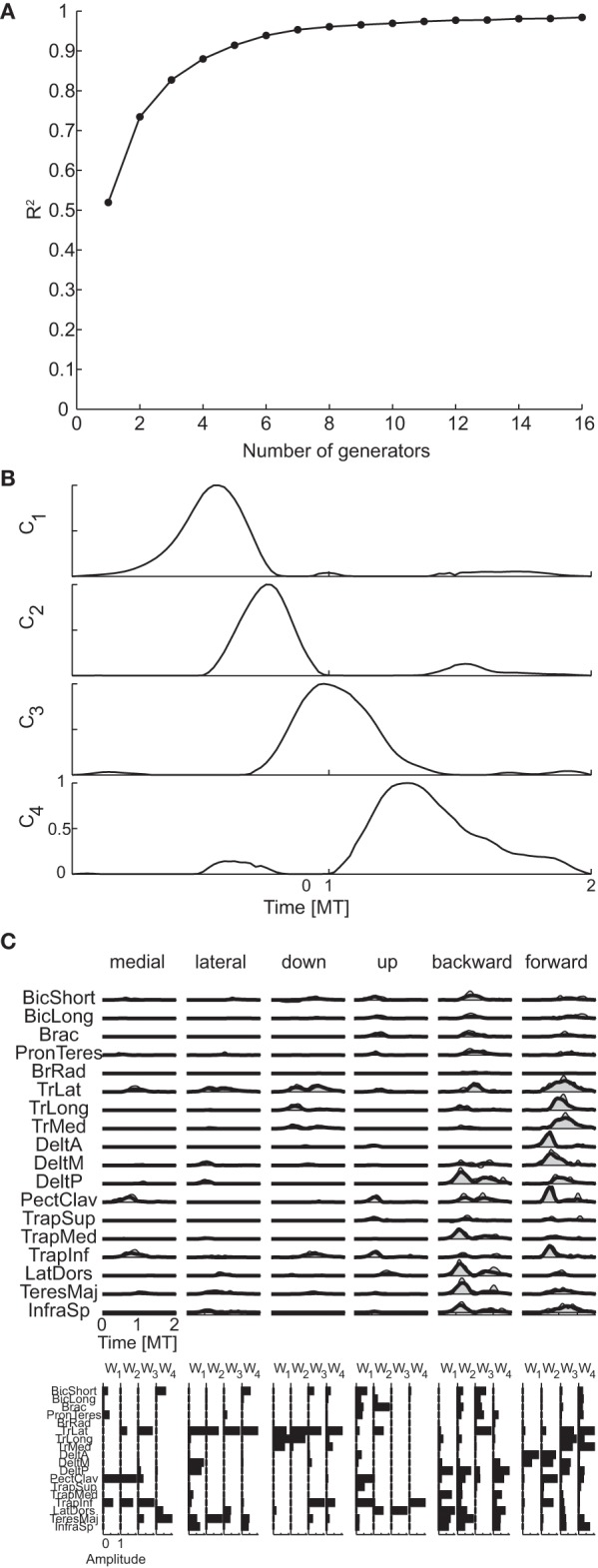
**Temporal decomposition of phasic muscle patterns. (A)** R^2^ curve for subject 1 obtained by temporal decomposition using NMF. **(B)** The four temporal generators selected for subject 1. **(C)** Example of the reconstructions of muscle patterns for six movement conditions of subject 1 obtained with the generator illustrated in panel **(B)** (*shaded area*: original data, *thick line*: reconstructed data, *bottom*: muscle-specific weights of each generator).

Figure 9A shows the R^2^ curve for the temporal decomposition of the phasic muscle patterns of subject 1 and Figure 9B the four temporal generators (or components) selected in that subject according to both criteria. The first three generators capture a single burst of muscle activity and the fourth component a small burst followed by a larger burst. The four components peak at different times and thus they appear to capture four distinct phases of the muscle patterns observed in different directions. However, the examples of muscle pattern reconstructions and combination weights for six movement directions (Figure 9C) show that in many cases the weight vectors loading the different components were similar within each movement condition (e.g., for the first two components of the medial movement and the last two components of downward movement), suggesting that such temporal decomposition was necessary to capture not only the major changes in the muscle patterns over the duration of the movement but also small asynchronous adjustments.

In contrast to the spatial generators but similarly to the temporal generators for torques, muscle pattern temporal generators were similar across all subjects. The *R*^2^-values for the reconstruction of the data of each subject by generators of all other subjects (0.82 ± 0.04, mean ±*SD*) were close to the values for the reconstruction by the generators extracted from the same data (0.85 ± 0.03) and higher than the *R*^2^-values obtained with generators identified from randomly shuffled data.

#### Spatiotemporal dimensionality

The mean number of spatiotemporal generators of the phasic muscle patterns was 5.5 according to the R^2^ knee criterion and 7.25 according to the R^2^ shuffle criterion (see Table 3 for individual values). Both dimensionality estimates were larger than the minimum number of generators required for generating a space with the same linear dimensions as the number of torque spatiotemporal generators (2.75) by non-negative combinations (3.75 = 2.75 + 1). Moreover, differently from torques, the product of the spatial and temporal muscle pattern dimensionalities (22.5 according to the R^2^ knee criterion and 18.7 according to the R^2^ shuffle criterion) was much higher than the spatiotemporal dimensionality. Thus the spatiotemporal generators captured asynchronous muscle coordination patterns that were not simply the result of the synchronous combination of all possible spatial and temporal generators.

**Figure 10:**
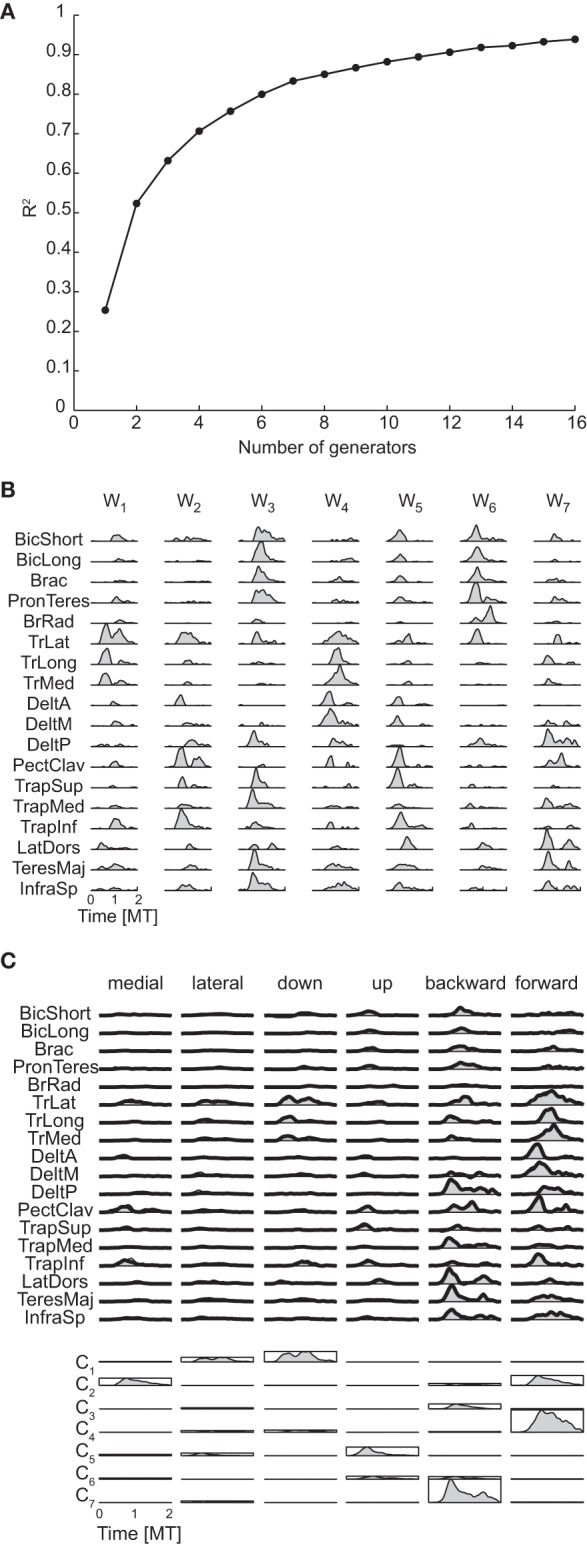
**Spatiotemporal decomposition of phasic muscle patterns. (A)** R^2^ curve for subject 1 obtained by spatiotemporal decomposition using NMF. **(B)** Seven spatiotemporal generators selected for subject 1. **(C)** Example of the reconstructions of the muscle patters for six movement conditions of subject 1 obtained with the generator illustrated in panel **(B)** (*shaded area*: original data, *thick line*: reconstructed data, *bottom*: combination coefficients represented by the height of the rectangle containing the temporal profile of each generators averaged over muscles).

Figure 10A shows the R^2^ curve for the spatiotemporal decomposition of the phasic muscle patterns of subject 1 and Figure 10B the seven spatiotemporal generators selected in that subject according to the R^2^ shuffle criterion. The asynchronous nature of the muscle activation waveforms can be noticed in most of these spatiotemporal generators. For example, in TrLat and TrLong in **w**_**1**_show two clearly delayed peaks. Finally, the examples of muscle pattern reconstructions and combination coefficients for six movement conditions illustrate how the organization of the muscle patterns is captured parsimoniously by the spatiotemporal generators as each movement is reconstructed specifying only 7 scalar combination coefficients (represented by the height of the rectangles depicting the mean generator waveform over all muscles).

Finally, muscle patterns of different subjects did not have similar spatiotemporal generators. The reconstruction of the data by generators of all other subjects had a much lower *R*^2^-values (0.37 ± 0.08, mean ±*SD*) than the *R*^2^-values for the reconstruction of the data by generators extracted from the same dataset (0.80 ± 0.03) but still higher than the *R*^2^-values obtained with generators identified from randomly shuffled data.

#### Effect of setting to zero negative values in the phasic muscle patterns

To identify muscle pattern generators from phasic muscle patterns using NMF we set to zero all negative values resulting from the subtraction of the tonic muscle activity from the filtered EMG waveforms. However, to assess the effect of such procedure we also extracted muscle pattern generators from the unclipped phasic muscle patterns using a gradient descent iterative algorithm (see Materials and Methods). In all cases the dimensionality of the generators identified with the gradient descent algorithm from the unclipped data was close to the dimensionality of the generators identified by NMF from the clipped data and the generators extracted in the two cases were very similar. The dimensionality of the spatial generators identified from the unclipped data was, on average across subjects, 4.75 (according to both R^2^ knee and R^2^ shuffle criteria) and thus differed only by 0.25 from the dimensionality of the generators identified from the clipped data. For the temporal generators the difference in dimensionality was 0.5 according to the R^2^ knee criterion and 0.25 according to the R^2^ shuffle criterion. Finally, for the spatiotemporal generators the difference was 0.25 according to both criteria. The similarity between the spatial generators identified from the clipped data and the same number of generators identified from the unclipped data, quantified by the mean normalized scalar product between matched pairs of generators, was 0.91 ± 0.09 (mean across subjects ± *SD*). For all subjects the similarity value was significantly higher that the value expected by chance, i.e., it was above the 95% percentile of the distribution of the similarity values between generators identified from the unclipped data and generators identified from the shuffled clipped data. The similarity between the temporal generators identified from the clipped data and the same number of generators identified from the unclipped data was 0.91 ± 0.05, also significantly higher than chance for all subjects. Finally, for spatiotemporal generators the similarity was 0.90 ± 0.02 and also significantly higher than chance for all subjects. We can thus conclude that clipping to zero the negative values of the phasic muscle patterns affected the dimensionality and the structure of the identified muscle pattern generators only minimally.

## Discussion

We assessed the dimensionality of the dynamic joint torques responsible for accelerating and decelerating the arm during point-to-point reaching movements in different directions in the frontal and sagittal planes and the dimensionality of phasic muscle patterns underlying the production of those torques. We used multidimensional factorization techniques, PCA for the torques and NMF for the muscle patterns, to identify generators capturing the spatial, temporal, and spatiotemporal organization of the motor commands. The number of generators selected according to either a threshold in the total data variation explained, or a change in slope in the curve of the variance explained, or the increase in data variation explained adding an additional generator with respect to the increase obtained extracting generators from randomly shuffled data was taken as an estimate of the dimensionality. The spatial dimensionality of the dynamic torques was lower than the number of joints considered, indicating that some of the available spatial coordination patterns were never employed by the CNS when generating the joint torques for this task. A single temporal generator with a biphasic activation profile was identified in all subjects, in accordance and generalizing previous observations on the temporal organization of dynamic torques on a single vertical plane (Gottlieb et al., 1997). However, a higher number of temporal generators may be required to account for more complex changes in joint torques in other types of reaching movements (e.g., slow, egocentric etc., see Lacquaniti et al., 1986). The number of spatiotemporal generators was in most subjects equal to the product of the spatial and temporal dimensionality and their structure indicated that the spatiotemporal organization of the dynamic torques was essentially synchronous, obtained by the temporal modulation of the spatial generators by the biphasic profile of the single temporal generator. In contrast, the spatial, temporal, and spatiotemporal dimensionalities of the phasic muscle patterns were higher than the corresponding torque dimensionality, as expected because of the non-negativity constraints in the combination of muscle pattern generators, but also higher than the minimum number required according to this biomechanical constraint. Moreover, the spatiotemporal dimensionality of the muscle patterns was much lower than the product of their spatial and temporal dimensionality, suggesting that specific asynchronous coordination patterns were used in the generation of muscle patterns. In fact, most of the identified spatiotemporal generators showed peaks of activity in different muscles at different times, i.e., coordination patterns that cannot be captured by the synchronous modulation of one of the spatial generators by one of the temporal generators.

The CNS might generate motor commands by organizing a few generators, basic elements in a modular architecture capturing shared knowledge across tasks and conditions, to reduce the number of parameters required for control (Alessandro et al., 2013; Ruckert and d'Avella, 2013). Evidence for a modular organization of the motor commands has recently come from the observation of low-dimensionality in the muscle patterns recorded in many species, behaviors, and tasks (Tresch et al., 1999; d'Avella et al., 2003, 2006; Hart and Giszter, 2004; Ivanenko et al., 2004; Ting and Macpherson, 2005; Overduin et al., 2008; Muceli et al., 2010; Dominici et al., 2011; Berger et al., 2013) and from neural recordings and stimulation (Saltiel et al., 2001; Ethier et al., 2006; Gentner and Classen, 2006; Hart and Giszter, 2010; Overduin et al., 2012). Intramuscular recordings during isometric contractions have also revealed that the number of basic muscle activation patterns in complex movements is very limited (ter Haar Romeny et al., 1984; van Zuylen et al., 1988). However, motor task and behaviors are accomplished by the joint torques generated by the simultaneous and coordinated activation of many muscles and to understand how a small set of muscle pattern generators may accomplish a task it is necessary to understand the relationship between muscle patterns and joint torques. The transformation between muscle patterns and torques depends on several biomechanical characteristics and constraints. There are more muscles than joints, making motor commands at the level of muscle patterns redundant, i.e., the same torque pattern can be generated by different muscle patterns. Muscles can only pull and their activation can be expressed by non-negative values, thus introducing a fundamental non-negativity constraint in the generation of muscle patterns. These characteristics and constraints affect the potential dimensionality of the joint torques associated to the dimensionality of the underlying muscle patterns. For a linear mapping of muscle activity into force, an assumption that may be true only in specific conditions such as submaximal isometric contractions (Borzelli et al., 2013) but useful for illustrative purposes, because of the non-negativity constraint, at least *D* + 1 generators are required to span a *D* dimensional torque space (Davis, 1954; Valero-Cuevas, 2009). In fact, by linearity, the image of the pseudo-inverse transformation of the *D* dimensional torque space is a *D* dimensional subspace of muscle space but such subspace is not contained in the positive orthant of the muscle space, i.e., cannot be generated by non-negative activations and an additional dimension in the null space of the linear transformation needs to be used to achieve a non-negative muscle pattern for each torque. In the general non-linear case, the manifold in muscle space containing all the minimum patterns associated to a *D* dimensional torque space may be already higher than *D* even before considering the non-negativity constraint. Thus, the dimensionality of the muscle space must be at least *D* + 1 to generate a *D* dimensional torque space for the non-negativity constraint and possibly larger. However, because of redundancy, the CNS might use a number of muscle pattern generator larger than the minimum to optimize some other cost in addition to the number of control parameters, such as effort. Since the generation of muscle patterns with a smaller number of generation requires in general more effort, the dimensionality of the muscle pattern generators might result from a trade-off between computational complexity and effort. We found that the muscle pattern dimensionality is indeed larger than the minimum prescribed by non-negativity, likely an effect of non-linearity but possibly also due to a choice of generators capable of achieving the same motions with less effort. Future investigations comparing tasks with similar kinematics but different effort might help clarify this point.

As mentioned in the Introduction, muscle pattern generators can be associated to force-field primitives (Bizzi et al., 1991; Giszter et al., 1993; Kargo and Giszter, 2000a,b; Giszter and Hart, 2013), endpoint force or joint torque generators that depend on joint angles and velocities and are linearly combined using the activation coefficients of the muscle pattern generators. Competence of force-field primitives to generate observed force and kinematic behaviors has been demonstrated in the frog through forward biomechanical simulation (Kargo et al., 2010). Similarly, a forward dynamics simulation of a musculoskeletal model of the human leg has shown that the combination of a small number of muscle pattern generators is sufficient to perform the basic sub-tasks of walking (Neptune et al., 2009). Moreover, a recent simulation study using a musculoskeletal model of the human arm has indicated that spatiotemporal generators adequate to perform reaching movements can be learned through reinforcement (Ruckert and d'Avella, 2013). Here, in contrast, we did not perform forward dynamics simulation to assess the competence of muscle pattern generators and associated force-field primitive to control reaching movements. We focused, instead, onto the dimensionality of recorded muscle patterns and the dimensionality of joint torques estimated from recorded kinematics by inverse dynamics. Force-field primitives are associated one-to-one with muscle pattern generators, i.e., they have the same dimensionality. Because of the non-negativity of muscle pattern generator activation coefficients and the non-linearity in the muscle-to-force mapping, such dimensionality is necessarily higher than the dimensionality of the joint torques, i.e., the number of torque generators necessary to adequately reconstruct the observed torques by linear combinations. As different numbers of muscle pattern generators and force-field primitives can potentially generate the same set of observed torques and thus being equally competent to perform a given behavior, comparing the dimensionality of muscle patterns and joint torque provides additional information on the strategy that the CNS employs to organize a modular control architecture.

We assessed the dimensionality of joint torques and muscle patterns according to three different definitions of generators (spatial, temporal, and spatiotemporal) and we could then also compare, within each dataset, the different types of dimensionality. For the torques we found that the dimensionality of the spatiotemporal generators was equal to the product of the dimensionalities of the spatial and temporal generators, suggesting that such generators can be obtained as the product of spatial and temporal generators (Delis et al., 2014). The spatial dimensionality was two or three, i.e., less than the number of angular DoF involved in the task, indicating that the CNS selected specific coordination strategies already at the kinematic level. The temporal dimensionality was one, indicating, in accordance with previous observations (Gottlieb et al., 1997), that the temporal organization of the dynamic torques is very simple: a bi-phasic profile shared by all joints and movement conditions (but see Lacquaniti et al., 1986 for more complex torque profiles). The dimensionality of the spatiotemporal generators was in all subjects equal to the spatial dimensionality because, given a single temporal generator, each spatiotemporal generator was obtained by the temporal modulation of each spatial generator by the temporal generator. Consequently, the spatiotemporal organization of the torques was essentially synchronous. In contrast, the number of spatiotemporal generators for the muscle patterns was much less than the product of spatial and temporal dimensionalities. Indeed, spatiotemporal generators captured asynchronous activations across muscles that could not be obtained by the modulation of a single spatial generator by a single temporal generator, which necessarily produces a synchronous pattern. Thus, spatiotemporal generators appear to provide a very compact representation of the organization of the muscle patterns (Delis et al., 2014). However, differently from our previous analyses of muscle patterns during reaching (d'Avella et al., 2006, 2008, 2011; d'Avella and Lacquaniti, 2013), in the present spatiotemporal decomposition we did not take into account the possibility of shifting in time the onset of different generators (Equation 8) because in this way we could use the same NMF algorithm used for spatial and temporal decomposition. We found a larger number of generators without time-shifts than the number of time-varying muscle synergies (with time-shifts) reported before. Thus, additional structure in the muscle patterns can be captured by allowing the independent modulation of the time of recruitment of the generators thus allowing for an even more compact representation of the muscle pattern organization.

The validity of our observations depends on a number of assumptions made in the analysis of the torque and muscle activity data. Concerning the estimation of the joint torques from the recorded motions of markers positioned on the subjects' arm, we relied on a simplified model of the human arm. We assumed that the shoulder was a spherical joint, i.e., all three rotation axes intersect at a single point, we estimated the length (Winter, 1990) and inertial parameters (Zatsiorsky and Seluyanov, 1983) of each segment as a function of the height and weight of each subject. To assess the effect of potential inaccuracies in the parameters of our model, we performed inverse dynamics varying the mass and height of each subject up to ±20% and we found that the estimated joint torque dimensionality changed only in a small fraction of cases. Thus, we believe that the estimation of torque dimensionality is robust to small inaccuracies in the anthropometric parameters. Concerning the identification of the muscle pattern generators with NMF, in order to be able to run such algorithm, we set to zero all samples with a negative value after subtracting the tonic components. We assessed the effect of such procedure by identifying muscle pattern generators from unclipped data using a gradient descent algorithm and we found that both the dimensionality changed only minimally (less than 0.5 in all cases) and did not significantly affect the structure of the generators. Concerning the criteria for the selection of the number of generators, we used a threshold based criterion for torques, a criterion based on the detection of a change in slope (i.e., a “knee”) in the R^2^ curve for muscle patterns, and a criterion based on the comparison of the slope of the R^2^ curve for the generators extracted from the original data and those extracted after randomly shuffling the data (along the rows of the data matrix). All these criteria rely on the assumption that the data are generated by a number of generators smaller than the maximum dimensionality and that a fraction of the variation observed in the data is due to noise. Such assumption is shared by all previous studies using multidimensional decomposition to identify muscle synergies or temporal components but it is clear that it is not possible a-priori to exclude that, once a specific number of generators has been selected, the additional dimensions attributed to noise might be also necessary to capture the structure in the motor commands or, vice-versa, a generator might actually describe noise instead of structure in the motor commands. Moreover, unless an independent estimation of the level of noise in the data is available, the selection of the number of generators depends on *ad-hoc* choices of thresholds and parameters. However, as the determination of threshold on the MSE of the linear fit of the terminal portion of the R^2^ curve, used for the R^2^ knee criterion, is less dependent on the amount noise in the data than the threshold on the *R*^2^-value, used in the R^2^ threshold criterion, we prefer, whenever possible, to use the former criterion, as we have done previously (d'Avella et al., 2003, 2006; Cheung et al., 2005), to the latter, also used in previous studies (Tresch et al., 1999; Ting and Macpherson, 2005; Torres-Oviedo et al., 2006; Roh et al., 2012). Unfortunately it was impossible to use the R^2^ knee criterion for the spatial torque dimensionality, as the maximum dimension was 4 in that case, and we could only estimate the residual of a linear fit of the R^2^ curve from 1 or 2 to 4 generators, i.e., evaluating the presence of a knee only at 1 or 2 generators. We then used the third criterion to compare more directly both datasets. The R^2^ shuffle criterion (Cheung et al., 2009) is based on the assumption that the multidimensional structure but not the noise is affected by randomly shuffling the data. Thus, the selection of the number of generators in datasets with different amount of noise is based on the comparison of the slope of the R^2^ curve for each data set with the slope of the R^2^ curve of a random dataset with comparable level of noise. In most cases the number of generators selected by the two criteria either matched exactly or different by one.

In conclusion, whether spatial (time-invariant muscle synergies), temporal (temporal components or patterns), or spatiotemporal (time-varying muscle synergies) generators are fundamental building blocks in a modular control architecture and how are they implemented in the CNS remain open and debated questions. Our comparison of the dimensionality of muscle patterns and joint torques suggests that the larger dimensionalities and spatiotemporal complexity of the muscle patterns with respect to the joint torques may be required for the CNS to overcome the non-linearities of the musculoskeletal system and, exploiting its redundancy, to flexibly generate endpoint trajectories with simple kinematic features using a limited number of building blocks.

### Conflict of interest statement

The authors declare that the research was conducted in the absence of any commercial or financial relationships that could be construed as a potential conflict of interest.
